# The Effect of 5G Mobile Phone Electromagnetic Exposure on Corticospinal and Intracortical Excitability in Healthy Adults: A Randomized Controlled Pilot Study

**DOI:** 10.3390/brainsci15111134

**Published:** 2025-10-22

**Authors:** Azadeh Torkan, Maryam Zoghi, Negin Foroughimehr, Shapour Jaberzadeh

**Affiliations:** 1 Monash Neuromodulation Research Unit, Department of Physiotherapy, School of Primary and Allied Health Care, Monash University, Melbourne, VIC 3800, Australia; azadeh.torkan@monash.edu; 2 Discipline of Physiotherapy, Institute of Health and Wellbeing, Federation University, Melbourne, VIC 3350, Australia; m.zoghi@federation.edu.au; 3 6G Research and Innovation Lab, Swinburne University of Technology, Melbourne, VIC 3122, Australia; nforoughimehr@swin.edu.au

**Keywords:** 5G, brain excitability, transcranial magnetic stimulation, motor-evoked potentials, mobile phone exposure, intracortical excitability

## Abstract

Background: Research on the impact of 5G mobile phone electromagnetic exposure on corticospinal excitability and intracortical mechanisms is still poorly understood. Objective: This randomized controlled pilot study explored the effects of 5G mobile phone exposure at 3.6 GHz (power density: 0.0030 W/m^2^) on corticospinal excitability and intracortical mechanisms in healthy adults. Methods: Nineteen healthy participants (mean age: 36.5 years) were exposed to 5G mobile phone exposure for 5 and 20 min, approximating the typical duration of a phone call. Corticospinal excitability, intracortical facilitation, short intracortical inhibition, and long intracortical inhibition using single- and paired-pulse transcranial magnetic stimulation assessed before and immediately after exposure were performed. Results: A two-way repeated-measures ANOVA revealed no significant interactions between exposure condition (5 min, 20 min, sham) and time (pre vs. post) for CSE, ICF, SICI, or LICI (all *p* > 0.15). Bayesian analyses yielded Bayes factors close to 1, indicating inconclusive evidence for both the null and alternative hypotheses. Conclusion: Short-term exposure to 5G mobile phone electromagnetic fields did not produce detectable changes in corticospinal or intracortical excitability. Bayesian evidence was similarly inconclusive (Bayes factors ≈ 1), suggesting that the data provide limited support for either the presence or absence of a detectable effect. Any potential influence of 5G exposure on neural function is therefore likely to be subtle with the present methods. As a pilot study, these findings should be interpreted cautiously and underscore the need for further research using more sensitive outcome measures, extended exposure durations, and vulnerable populations.

## 1. Introduction

### 1.1. Background

#### 1.1.1. Evolution of Mobile Phone Technology and Electromagnetic Radiation

Fifth-generation (5G) mobile phone (MP) technology, first introduced globally in 2019, uses higher-frequency electromagnetic radiation (EMR) than earlier generations [1,2]. The operation of (MPs) relies on EMR, which propagates as waves traveling at 3 × 10^8^ m/s in free space [1].

MPs emit EMR mainly as radiofrequency radiation (RFR), a form of non-ionizing radiation that lacks sufficient energy to ionize atoms or molecules [3]. RF operates within the range of 100 kHz to 300 GHz [4], transmitting signals between mobile devices and cellular towers to enable wireless communication. RF waves are part of the larger electromagnetic (EM) spectrum, which encompasses a broad range of frequencies, from low-frequency waves, such as those associated with mobile phone EM exposure, to high-frequency waves, including X-rays [5]. 5G operates at higher frequencies using shorter millimeter waves than earlier generations, such as 4G, across low-band (600–900 MHz), mid-band (1.7–4.7 GHz), and high-band (24–47 GHz) frequencies [6,7,8,9,10]. The transition from 2G to 5G networks represents a significant advancement, offering faster data speeds, reduced latency, and enhanced connectivity, thereby improving network performance [11]. This widespread adoption, facilitated by expanded telecommunication networks, has resulted in increased exposure to EMR, mainly an increase in the number of MP users rather than a naturally harmful increase in EMR levels.

#### 1.1.2. Neurophysiological Implications of Mobile Phone EM Exposure

Fifth-generation mobile phone EM exposure involves higher frequencies and shorter wavelengths than earlier generations, which may influence neural excitability. It is essential to examine key neural mechanisms, such as corticospinal excitability (CSE) and intracortical excitability, especially given that MPs are typically held close to the head.

CSE reflects the responsiveness of pyramidal neurons and descending motor pathways that transmit signals from the motor cortex to spinal motor neurons [12,13,14], forming the foundation of voluntary motor control [15,16]. In contrast, intracortical excitability refers to the balance of excitatory and inhibitory interactions among cortical neurons, mediated by facilitatory and inhibitory interneurons. Both CSE and intracortical excitability can be assessed using transcranial magnetic stimulation (TMS), which investigates specific intracortical mechanisms including intracortical facilitation (ICF) [17], long-interval cortical inhibition (LICI) [18], and short-interval cortical inhibition (SICI) [19]. This approach aligns with established methods in environmental neurophysiology and cognitive neuroscience for examining the cortical effects of RF-EMFs.

These neurophysiological markers offer different insights into the brain’s excitation-inhibition balance [20,21,22], which underpins various functions, including motor learning, attention, and fatigue recovery [20,21,22,23,24,25]. For example, increased ICF, which is mediated by glutamatergic transmission, reflects greater cortical excitability and responsiveness, often linked to improved sensorimotor integration [26,27,28]. Conversely, reduced SICI, mediated by GABA-A neurotransmission, may suggest decreased inhibitory control, potentially leading to increased cortical noise or decreased motor precision [29,30,31]. LICI, mediated by GABA-B receptors, reflects both fast and slow inhibitory processes in the motor cortex [32]. CSE, which reflects the net output from the motor cortex via the corticospinal tract, serves as an indicator of the excitability of pyramidal neurons and spinal motor neuron pathways [33]. CSE is known to vary in response to factors like motor learning, fatigue, and neurological conditions [34,35]. ICF, SICI, and LICI are all mediated by different neurotransmitter systems, providing a layered understanding of cortical excitability and regulation [35,36,37]. Their modulation has real-world implications for motor function, cognition, and health outcomes, making them critical markers for assessing potential neurophysiological impacts of EMR.

#### 1.1.3. Gaps in Current Research

The effects of mobile phone EM exposure on brain oscillations, particularly during 3G and 4G use, have been extensively studied with electroencephalography (EEG) [38,39,40]. Previous studies on 3G/4G mobile phone EM exposure using EEG consistently report an increase, rather than a decrease, in brain oscillatory activity, especially within the alpha and beta frequency bands [41]. These changes demonstrate the sensitivity of cortical networks to EM fields, mainly in oscillatory domains. However, EEG oscillations reflect different neural processes compared to TMS-based measures [42,43,44,45]. While EEG captures rhythmic synchronization, TMS evaluates cortical excitability and intracortical dynamics using RMT, CSE, SICI, ICF, and LICI. These neural markers reflect different inhibitory and facilitatory mechanisms within the motor cortex (M1). Advances in technology have introduced 5G, which utilizes higher-frequency millimeter-wave (mmWave) signals. These signals primarily interact with superficial tissues and may stimulate sensory nerve endings in the skin [46,47], raising concerns about thermal and non-thermal effects on the nervous system. A knowledge gap exists regarding the impacts on cortical processes, such as CSE, ICF, SICI, and LICI. EEG suggests sensitivity to EM fields; however, the effect of 5G on CSE, as measured by TMS, remains unknown. This clarifies our hypotheses and study design. Despite growing interest, the understanding of 5G radiation’s impact on CSE and intracortical excitability in motor and non-motor regions is limited.

To date, only two studies (Table 1) have investigated the effects of mobile phone EM exposure on brain excitability [48,49]. Both used comparable TMS-based approaches but differed in exposure duration, participant demographics, and device specifications. While [48] reported increased ICF and reduced SICI, [49] found no significant effects on CSE or SICI. Neither study examined LICI.

Existing studies have methodological limitations, like a lack of diversity in volunteer age and gender, inadequate power analysis, and insufficient focus on exposure distance from the head, a crucial variable. Considering the varying distances in experimental design is vital. While evidence suggests cortical sensitivity to EM fields, it remains unclear whether 5G exposure affects excitability, as measured by TMS. Addressing these gaps is crucial to understanding the neurophysiological effects of new technologies.

#### 1.1.4. Significance of Current Research

The rapid adoption of 5G MP technology has raised concerns about the possible health effects of its higher frequencies [8,47], particularly concerning neural mechanisms and prolonged use of mobile devices near the head. CSE and intracortical excitability are vital for motor control, cognitive performance, and sensory integration. Investigating how RFR exposure, especially from 5G technology, affects these mechanisms is essential because they underpin key brain functions. Previous studies have raised concerns about the potential effects of MPs use on cognition, visuomotor coordination [50], reaction time [51,52], and balance control [53], along with reports of subjective symptoms like headaches, localized pain, and discomfort near the exposure site [54,55,56,57,58]. Research on the effects of 5G radiation on CSE and intracortical excitability remains limited, highlighting the need for comprehensive studies to fill these gaps and enhance our understanding of 5G’s neurophysiological impact. The findings will support the development of public health guidelines, increase consumer awareness, and enable informed decisions about 5G use, balancing its advantages with potential health risks.

This study is presented as an exploratory pilot, aiming to address these gaps by investigating the neurophysiological effects of 5G mobile phone EM exposure:

#### 1.1.5. Study Aims

1. To determine whether a 5 min exposure to 5G mobile phone EM exposure significantly affects CSE and associated inhibitory and facilitatory mechanisms (ICF, SICI, and LICI) compared to a sham condition.
2. To assess whether a more prolonged exposure duration (20 min) differentially influences CSE and these mechanisms, compared to both 5 min exposure and sham conditions.

To systematically examine the neurophysiological effects of 5G EM exposure and address the goals while filling the gaps in the limited existing evidence in this domain, the present study was structured around the following hypotheses.

#### 1.1.6. Hypotheses

1. Both 5 min and 20 min exposures to 5G mobile phone EM radiation will cause significant changes in CSE and intracortical excitability (ICF, SICI, and LICI) compared to a sham exposure.
2. A 20 min 5G EM exposure will produce greater modulation of CSE and intracortical excitability (ICF, SICI, LICI) compared with both the 5 min and sham conditions, consistent with a duration-dependent effect.

## 2. Materials and Methods

### 2.1. Participants

Nineteen healthy volunteers (10 females, 9 males; age range: 20–53 years; mean age: 36.5 years) participated in this study. Given the pilot nature of this study and its focus on feasibility and methodological development, the sample size was not sufficient to conduct subgroup analyses by sex or age. Such analyses should be pursued in future larger-scale investigations. The study was approved by the Monash University Human Research Ethics Committee (Approval No. 34411, 1 December 2022). All participants provided written informed consent in agreement with the Declaration of Helsinki. Participants were monitored for signs of distress or adverse reactions during and after the sessions and were encouraged to report any delayed effects.

### 2.2. Sample Size Justification

This study was conducted as a pilot study with the primary aim of assessing feasibility, refining experimental procedures, and generating preliminary data to inform future large-scale trials [59,60]. As such, a formal a priori power calculation was not the basis for determining sample size. Instead, the sample size (*n* = 19) was chosen pragmatically, considering feasibility and resource constraints, in line with recommendations for pilot studies [61] that typically recruit between 12 and 30 participants [62,63] to provide sufficient preliminary information. The data obtained here will be used to inform more accurate power calculations for adequately powered confirmatory studies [61,63].

### 2.3. Inclusion-Exclusion Criteria

Eligible participants were healthy, right-handed adults aged 18–60 years, with no risk factors for TMS as assessed by a standardized questionnaire [64]. Exclusion criteria included the following: history of brain injury; epilepsy in first-degree relatives; metallic implants in the head (excluding dental work); cardiac pacemakers or intracardiac lines; frequent or severe headaches or migraines; implanted neurostimulators; surgical clips; medical pumps; or other electronic medical devices. Individuals with neurological or psychological disorders, those using neuropsychotropic drugs, and pregnant individuals were also excluded. Participants were instructed to sleep at least 7 h, abstain from alcohol for 48 h, caffeine and energy drinks for 3 h, and dynamic exercise for 24 h before the experiment. Compliance was confirmed via self-report.

### 2.4. Study Design

**Figure 1:**
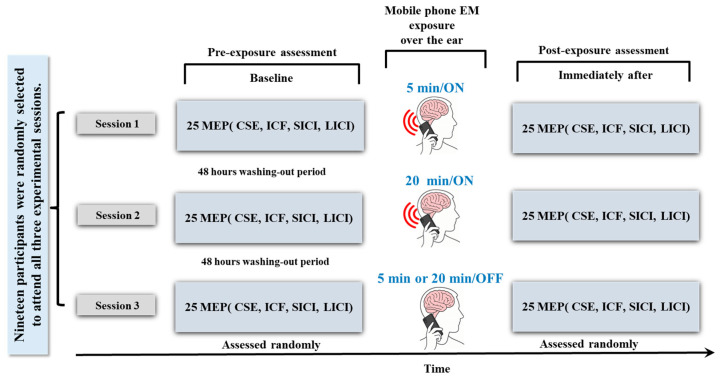
Overview of experimental design across all three experimental conditions (5 min, 20 min, and sham) for each outcome measure (CSE, SICI, LICI, ICF). CSE: Corticospinal Excitability; SICI: Short Intracortical Inhibition; LICI: Long Intracortical Inhibition; ICF: Intracortical Facilitation; MEP: Motor Evoked Potential.

A randomized, sham-controlled, crossover design was used, with a washout period of at least 48 h between sessions to mitigate carryover effects. All participants completed each of the three experimental conditions: (1) Five minutes of mobile phone EM exposure (talking mode); (2) Twenty minutes of mobile phone EM exposure (talking mode), and (3) Five or twenty minutes with a switched-off mobile phone (no exposure or sham). This ensured that every participant served as their own control. This format reduced variation between individuals and increased the consistency of comparisons across conditions. The order of condition administration was counterbalanced to control for potential order effects. Participants held the MP in their left hand, about 1 cm above the left ear, while seated in a relaxed posture during the experiment sessions. The order of these conditions was randomized across participants (Figure 1). Sessions were scheduled between 12:00 and 17:00 to minimize diurnal variation in cortical excitability. Exposure durations were selected to reflect common patterns of short-term MP use.

Participants engaged in a standardized verbal interaction with the experimenter, who was seated approximately two meters behind them during all three sessions. In the talking-mode condition, the “conversation” consisted of identical, scripted dialog (a predetermined list of questions and answers) that was similar for all participants and across all sessions. This ensured that the verbal interaction was fully standardized and consistent throughout the study. The phone was muted, so the only sound was the participant’s own voice, which remained the same in all conditions. This design controlled for potential dB(A)-related confounds while maintaining ecological validity, ensuring that exposure differences, not sound level, were the sole variable. This protocol was applied to control for the effects of social interaction and to standardize experimental procedures, ensuring that the duration of exposure was the only variable. In the sham condition, the same protocol was followed, but the MP was switched off to act as a control for non-RF-related effects.

This study used the 5G MP Galaxy A13, Model SM-A136B (Samsung Electronics Co., Lt., Suwon, Republic of Korea), to ensure consistency across three experimental conditions. All connections were in talking mode without data streaming and were supported by the Telstra provider in the Frankston area in southeast Melbourne. This specific model was selected based on its availability and representative characteristics of 5G MPs operating at a frequency of 3.6 GHz according to the Radio Frequency National Site Archive (RFNA). While this model limits the generalizability to other 5G devices, the purpose of this study is to assess the effects of 5G exposure on cortical and behavioral outcomes. Therefore, using a specific device with a known radiation profile provides a controlled environment for precise measurements.

### 2.5. Sham Intervention

In sham sessions, participants held the same phone in the identical position, but it was switched off to eliminate EM exposure. This controlled for sensory and positional influences, although expectancy or placebo effects could not be entirely ruled out.

### 2.6. Exposure Setting

#### 2.6.1. Device Specifications and EMR Compliance

The Galaxy A13 5G, operating at a primary frequency of 3.6 GHz, was chosen for its representativeness of mid-band 5G devices. According to data from the Radio Frequency National Site Archive, the EMR level at this site, as reported by the Australian Radiation Protection and Nuclear Safety Agency (ARPANSA), was 2.20% of the public exposure limit at a distance of 46 m from the location. Measurements of the site’s EMR were based on publicly available data (Radio Frequency National Site Archive, n.d.). However, the radiation values reported in this study (e.g., power density (PD) = 0.0030 W/m^2^, electric field (E-field) = 1.5 V/m) were obtained from direct real-time measurements conducted in the laboratory using a Narda EMR-300 radiation meter during active handset operation.

The phone operated across a combination of low- and high-band frequencies for both 4G (0.7 GHz, 1.8 GHz, and 2.1 GHz) and 5G (2.6 GHz, 0.85 GHz, and 3.6 GHz). In Australia, the Australian Communications and Media Authority (ACMA) regulates communication and media services, including the operation of 5G across different frequency bands [8].

Low-band 5G, which operates below 1 GHz, offers broader coverage and improved building penetration; however, it is accompanied by reduced speed and capacity. Mid-band 5G, operating within the 1–6 GHz range, strikes a balance among coverage, penetration, and network performance. Meanwhile, high-band 5G, also known as mmWave, operates at frequencies around 26 GHz (specifically 25.1–27.0 GHz and above), supporting higher speeds and greater capacity; however, its range is shorter and its ability to penetrate buildings is limited. Due to these properties, the current study’s measurements and electromagnetic field assessments are particularly focused on low-band and mid-band 5G frequencies.

The MP used in the setup was transmitted at a frequency of 3.6 GHz, and each measurement was repeated five times before averaging. Background readings were taken both with all devices turned off and with them switched on, to check for any unintentional environmental electromagnetic interference (EMI), following ICNIRP’s recommended procedures.

Real-time measurements of PD and E-field were recorded during each experimental session, allowing for accurate monitoring of exposure levels. These recorded levels were significantly lower than the safety threshold outlined by the International Commission on Non-Ionizing Radiation Protection (ICNIRP) [47] and Institute of Electrical and Electronics Engineers (IEEE) [46] guidelines, which specify a maximum public exposure limit of 40 W/m^2^ for PD within the 2–6 GHz frequency range. During phone call scenarios, the observed PD was 0.0030 W/m^2^, while the average E-field reached 1.5 V/m, and the calculated E-field was 1.06 V/m, indicating full compliance with established health and safety standards. For comparison, ARPANSA reports that at a distance of 400 to 500 m from a base station (such as at Monash Frankston, where this study was conducted), the E-field is 2.4 V/m with a PD of 0.01644 W/m^2^.

Our lower readings are likely caused by different conditions, as ARPANSA’s Environmental Electromagnetic Energy (EME) data indicate the maximum field levels at monitoring sites [65]. Those reported values assume that every transmitter planned for a site is installed and operating at full power. In practice, though, many transmitters at a base station are only activated when call or data traffic increases; otherwise, they remain inactive. Even during an active call, the system adjusts transmission power to the lowest level required to maintain a stable connection. When a handset is nearby or in a strong signal zone, the base station automatically reduces its transmission power.

#### 2.6.2. Environmental EMR Control

To maintain consistent exposure settings and reduce background EMR levels across all experimental sessions, Wi-Fi, MPs, and Bluetooth devices were turned off. Additionally, to minimize external noise and prevent interference with CSE and motor-evoked potential (MEP) assessments, all potential RF sources were positioned away from the TMS and electromyography (EMG) systems in the lab.

### 2.7. Outcome Measures

CSE was assessed using single-pulse TMS to elicit MEPs recorded from the right first dorsal interosseous (FDI) muscle via surface EMG. The primary outcome was the peak-to-peak MEP amplitude, recorded at baseline and immediately after the intervention [15,66,67]. Neurophysiological measurements were conducted before and immediately after exposure, not during the active exposure period. This approach was chosen to prevent EMR interference between the 5G device and TMS recording equipment, ensuring data integrity. Additionally, measuring during exposure could have raised safety and comfort concerns for participants, such as coil heating and unstable signal acquisition. By recording immediately after exposure, we minimized these confounds while still capturing potential immediate aftereffects of 5G EMR [68,69,70].

Intracortical excitability was evaluated using paired-pulse TMS (ppTMS), measuring ICF, SICI, and LICI at the same time points.

### 2.8. TMS Assessment of CSE

**Figure 2:**
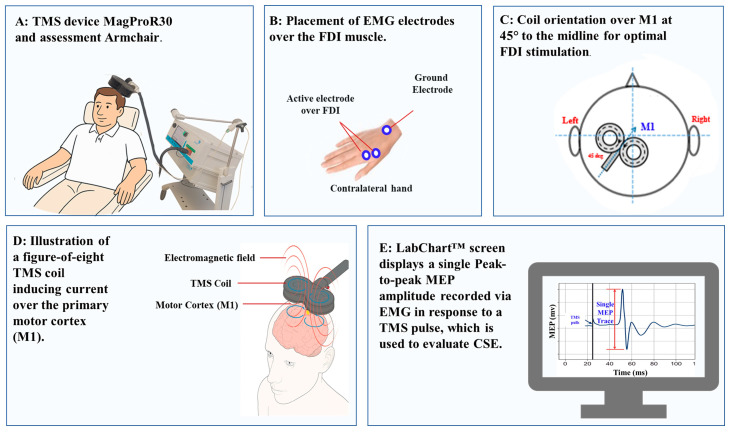
Experimental setup for evaluating CSE (Corticospinal Excitability) using TMS (Transcranial Magnetic Stimulation). (**A**) MagProR30 TMS device and adjustable assessment chair for positioning participants in an armchair; (**B**) Electromyography (EMG) electrodes placement over the first dorsal interosseous (FDI) muscle; (**C**) Orientation of the TMS coil over the primary motor cortex (M1) at 45° to the midline to optimize stimulation of the FDI representation; (**D**) Illustration of the induced current in the motor cortex generated by the electromagnetic field of the figure-eight TMS coil. (**E**) Representative motor evoked potential (MEP) trace recorded via EMG on LabChart™ (ADInstruments, Dunedin, New, Zealand), showing peak-to-peak amplitude in response to a single TMS pulse, used as the measure of CSE. ms: milliseconds; mV: millivolts.

Single-pulse biphasic TMS was delivered using a MagPro R30 stimulator (MagVenture, Farum, Denmark) and a figure-of-eight coil, positioned tangentially over the left motor cortex at a 45° angle (Figure 2A,C) to the midline [71,72]. The motor hotspot (M1) was identified as the scalp location (Figure 2C) eliciting the largest and most consistent MEPs from the right FDI (Figure 2E). The coil position was marked with a non-permanent marker to ensure consistency across sessions [71,73].

The resting motor threshold (RMT) was defined as the lowest stimulation intensity that evoked MEPs ≥ 50 µV in at least 5 of 10 trials in the relaxed FDI [16]. Test intensity was set to elicit ~1 mV MEPs before and after each session. A 6 s inter-stimulus interval minimized MEP variability [74,75]. In the present study, we chose to evoke MEPs at rest for the following reasons:

1. Standardization of CSE Assessment: Our primary aim was to examine the effects of 5G EMR on CSE and intracortical excitability under baseline (resting) conditions. Resting MEPs are widely used in TMS protocols to evaluate CSE, ICF, SICI, and LICI, without the confounding influence of peripheral or voluntary motor drive. This approach ensures that changes in MEP amplitude or inhibition/facilitation reflect cortical modulation, not task-related motor activation.
2. Avoidance of Variability from Contraction Effort: Sustaining even low levels of contraction (5–10% MVC) can introduce variability due to differences in attention, fatigue, or effort across time points and participants. Using the resting state minimizes such sources of variability and enhances reproducibility in pre–post comparisons.
3. Safety and Comfort: Resting MEPs reduce the physical demands placed on participants, which is particularly relevant given the repeated-measures design involving multiple TMS protocols across several sessions. This choice optimized participant comfort and compliance without compromising data quality.

### 2.9. TMS Assessment of Intracortical Excitability (ICF, SICI, LICI)

Paired-pulse transcranial magnetic stimulation (ppTMS) was used to evaluate intracortical excitability by delivering two consecutive magnetic pulses over the primary motor cortex (M1) [76]. The first pulse, known as the conditioning stimulus, was applied at a subthreshold intensity to modulate cortical excitability, while the second test stimulus was delivered at a suprathreshold intensity to elicit a measurable MEP. This method allows for the assessment of both inhibitory and facilitatory intracortical mechanisms [77]. The inter-stimulus interval (ISI) determines whether intracortical inhibition or facilitation occurs, affecting neural plasticity in healthy individuals and neurological patients [74,78].

**Figure 3:**
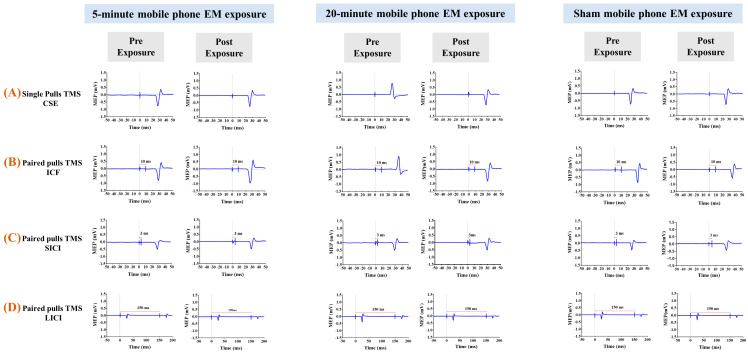
Representative raw data on mobile phone electromagnetic exposure effects, obtained from LabChart and replotted in Prism, showing typical recordings across all three experimental conditions (5 min, 20 min, and sham) for each outcome measure. Blue traces show single-trial MEPs recorded from the first dorsal interosseous (FDI) muscle. Red horizontal bars indicate interstimulus intervals (ISI) used in paired-pulse protocols: 3 ms for Short-interval Intracortical Inhibition (SICI), 10 ms for Intracortical Facilitation (ICF), and 150 ms for Long-interval Intracortical Inhibition (LICI). Corticospinal Excitability (CSE) was assessed using single-pulse TMS (no ISI). These traces provide raw data examples of the paradigms used in the study, illustrating how the Motor Evoked Potential (MEP) peak-to-peak amplitude was derived for each condition.

- Intracortical facilitation (ICF): ISI = 10 ms (Figure 3B). ICF is believed to involve excitatory glutamatergic transmission via NMDA and AMPA receptors, leading to an enhanced MEP response [79,80,81].

Three ppTMS protocols were applied, each targeting different neurophysiological pathways:

- Short interval intracortical inhibition (SICI): Inter-stimulus interval (ISI) = 3 ms (Figure 3C). SICI reflects inhibitory processes mediated by GABA A receptors, typically resulting in a suppressed MEP response compared to unconditioned pulses [19,82].
- Long-interval intracortical inhibition (LICI): ISI = 150 ms (Figure 3D). Both the conditioning and test stimuli were set at 120% of the RMT [74], and the resulting inhibition is thought to be mediated primarily by GABA B receptors [36,83,84,85,86].

For both SICI and ICF, the conditioning stimulus was set at 80% of RMT, while the test stimulus was adjusted to evoke MEPs of approximately 1 mV in peak-to-peak amplitude. These measurements were recorded at baseline and immediately following each experimental exposure session to assess potential changes in intracortical excitability. The use of standardized ISIs and intensities ensures reliable activation of the underlying inhibitory or excitatory circuits [19,80,84].

### 2.10. TMS Experimental Process

Participants were seated with their dominant hand supported on a pillow. Surface EMG was recorded from the right FDI muscle (Figure 2A). The skin in the target area was prepared with alcohol wipes and abrasive pads before placing bipolar Ag/AgCl electrodes following SENIAM guidelines. Bipolar Ag/AgCl electrodes were placed 2 cm apart on the muscle belly, with a reference electrode on a nearby bony landmark (Figure 2B). Signals were filtered (10–500 Hz), amplified (×1000), and sampled at 1000 Hz.

RMT was re-evaluated before each session. For single-pulse TMS (assessing CSE), a test stimulus at 120% of the pre-intervention RMT was used to record 25 MEPs per time point; this intensity was maintained for direct comparability. For paired-pulse TMS (assessing SICI, ICF, LICI), RMT was reassessed both pre- and post-intervention. The post-intervention RMT was used to recalibrate intensities: for SICI and ICF, the conditioning stimulus was 80% RMT with the test stimulus adjusted to evoke ~1 mV MEPs; for LICI, both pulses were at 120% RMT. Twenty-five conditioned and unconditioned MEPs were recorded per protocol.

Each session comprised 100 randomized MEP trials (25 per protocol) with a fixed 6 s inter-trial interval. To control for confounds, participants abstained from alcohol and caffeine for 12 h before sessions, which were separated by 48 h, and assessments occurred at a consistent time of day [87].

### 2.11. Statistical Analysis

Statistical repeated-measures two-way ANOVA was conducted to examine the effects of mobile phone EM exposure conditions (5 min, 20 min, Sham) and time (Pre, Post) interaction using GraphPad Prism (v 10.1.2) [88]. A one-way repeated-measures ANOVA assessed baseline differences in RMT, CSE, ICF, SICI, and LICI across the three experimental conditions. All statistical analyses considered age as a covariate to control for individual variability due to the broad age range of participants. Data normality was assessed using the Shapiro–Wilk test and visual inspection of histograms after outlier removal. Outliers were identified using Prism’s built-in detection methods. Analyses were performed in a blinded manner.

Post hoc comparisons were conducted using Tukey’s multiple comparisons test to control the family-wise error rate across repeated contrasts. This adjustment takes into account the number of comparisons within each test family and yields adjusted *p*-values.

To complement the frequentist analyses, Bayesian repeated-measures ANOVAs were conducted using JASP (v 0.19.3) [89] to quantify the strength of evidence for the effects of condition, time, and their interaction. Bayesian inference uses Bayes Factors (BF) to compare the relative support for competing hypotheses, providing a graded measure of evidence (BF < 1 indicates evidence in favor of the null hypothesis). According to conventional interpretation [90,91], a Bayes Factor (BF_10_) close to 1 indicates inconclusive evidence rather than evidence in favor of the null hypothesis.

Additionally, Intraclass Correlation Coefficients (ICCs) were calculated using a two-way mixed-effects model in SPSS (v 29.0.2.0) [92] to evaluate the test–retest reliability of TMS outcome measures across sessions

## 3. Results

**Figure 4:**
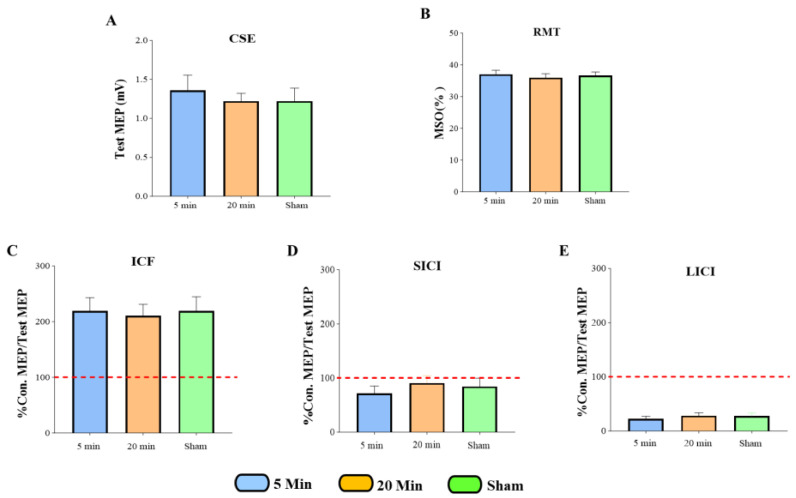
Statistical comparison of mobile phone electromagnetic exposure effects across all three experimental conditions (5 min, 20 min, and sham) on baseline assessments for outcome measures CSE (**A**), RMT (**B**), ICF (**C**), SICI (**D**), and LICI (**E**). The red dotted line at the ratio of 100% represents the baseline or control level of MEP amplitude. This line serves as a reference point, indicating the level of MEP response without any inhibitory or facilitatory conditioning stimulus applied. Con.: Conditioned, MEP: Motor Evoked Potentials, CSE: Corticospinal Excitability, ICF: Intracortical Facilitation, SICI: Short Interval Intracortical Inhibition, LICI: Long Interval Intracortical Inhibition, RMT: Resting Motor Threshold.

A one-way repeated-measures ANOVA was used to assess baseline differences in RMT, CSE, ICF, SICI, and LICI across the three experimental conditions (Figure 4). Descriptive statistics, including means and standard deviations, were calculated for each measure. The analysis revealed no significant differences in baseline values across conditions, indicating comparability before the intervention.

CSE: F (2, 54) = 0.2557, *p* = 0.7753; RMT: F (2, 51) = 0.1983, *p* = 0.8207, ICF: F (2, 36) = 0.05179, *p* = 0.9496; SICI: F (2, 36) = 0.6745, *p* = 0.5157; LICI: F (2, 36) = 0.6334, *p* = 0.5366.

These results show that baseline neurophysiological measurements were consistent across all experimental conditions. This consistency supports the conclusion that there were no carryover effects between sessions. To ensure this, a 48 h washout period was used between each session. Based on previous research [93], this time frame was sufficient to eliminate any remaining effects from earlier sessions. As a result, we can be confident that any changes observed after the interventions are not due to baseline differences but rather reflect the genuine effects of the experimental conditions.

An intraclass correlation coefficient (ICC) analysis was performed, further confirming the reliability of baseline ICC = 0.724 for CSE, ICC = 0.933 for RMT, ICC = 0.383 for ICF, ICC = 0.548 for SICI, and ICC = 0.617 for LICI measures across the baseline assessment.

### 3.1. The Effects of 5G Mobile Phone EM Exposure on CSE, ICF, SICI, and LICI

A two-way repeated-measures ANOVA was conducted with the factors of exposure condition (5 min, 20 min, sham) and time (pre, post) for each outcome measure. The analysis revealed no significant interaction effects between Exposure condition and Time in the 5 min and 20 min sessions for any outcome measures in CSE, like the sham condition.

CSE: F (2, 54) = 1.897, *p* = 0.1599; ICF: F (2, 54) = 0.4178, *p* = 0.6606; SICI: F (2, 54) = 0.5051, *p* = 0.6063; LICI: F (2, 54) = 1.720, *p* = 0.1887.

### 3.2. The Effects of 5G Mobile Phone EM Exposure on CSE

The two-way ANOVA revealed no significant interaction between exposure condition (5 min, 20 min, sham) and time (Pre, Post) for CSE (*p* = 0.1599). Mean CSE values remained consistent across all conditions.

**Figure 5:**
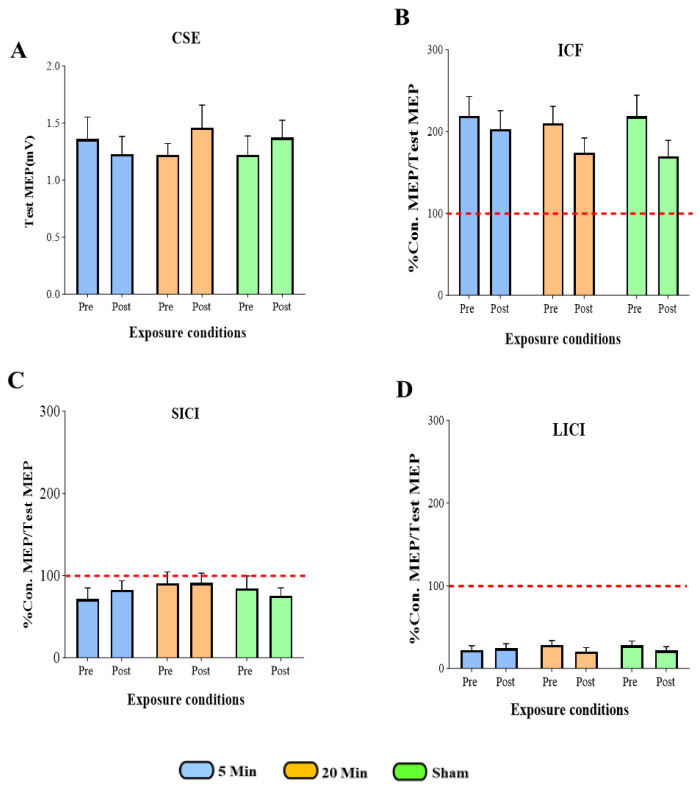
Statistical comparison of mobile phone electromagnetic exposure effects across three experimental conditions (5 min, 20 min, and sham) for each outcome measure: CSE (**A**), ICF (**B**), SICI (**C**), and LICI (**D**). The red dotted line at the ratio of 100% represents a baseline or control level of MEP amplitude. This line serves as a reference point, indicating the level of MEP response without any inhibitory or facilitatory conditioning stimulus applied. Con.: Conditioned, MEP: Motor Evoked Potentials, Test MEP (CSE): Corticospinal Excitability, ICF: Intracortical Facilitation, SICI: Short Interval Intracortical Inhibition, LICI: Long Interval Intracortical Inhibition.

Bayesian repeated-measures ANOVA yielded a Bayes Factor (BF_10_) of approximately 1.00 for the critical interaction term. A BF_10_ near 1 indicates that the data are inconclusive; they do not provide meaningful evidence to distinguish between the null hypothesis (no effect of 5G exposure) and the alternative hypothesis (an impact of 5G exposure). Although the null model had the highest posterior probability (*p* = 0.620), Bayes factors near 1 indicate that the data do not provide meaningful discrimination between the null and alternative models. Therefore, these results are best interpreted as inconclusive evidence for an effect of 5G exposure on CSE under the tested conditions (Figure 5A).

### 3.3. The Effects of 5G Mobile Phone EM Exposure on ICF

The two-way ANOVA showed no significant interaction between exposure condition (5 min, 20 min, sham) and time (Pre, Post) for ICF (*p* = 0.6606). Mean ICF values were comparable across conditions.

Bayesian repeated-measures ANOVA posterior probabilities of 0.450 for the time-only model and 0.387 for the null model, indicating that these models were nearly equally likely. Their corresponding Bayes Factors (BF_10_ = 1.164 for the time-only model; BF_10_ ≈ 1.00 for the null) were close to 1, reflecting inconclusive evidence. Models that included exposure conditions or interaction terms received negligible support (BF_10_ < 1). Taken together, these findings suggest that the data remain inconclusive regarding any effect of 5G exposure on ICF under the tested conditions (Figure 5B).

### 3.4. The Effects of 5G Mobile Phone EM Exposure on SICI

The two-way ANOVA showed no significant interaction between exposure condition (5 min, 20 min, sham) and time (Pre, Post) for SICI (*p* = 0.6063). Mean SICI values remained consistent across conditions, with no notable differences between exposures.

Bayesian repeated-measures ANOVA indicated that the null model had the highest posterior probability (*p* = 0.654), while the corresponding Bayes Factor (BF_10_ ≈ 1.00) was close to 1. This reflects inconclusive evidence, as the data were nearly equally compatible with both the null (no effect) and alternative hypotheses. Models including time (BF_10_ = 0.249) or exposure (BF_10_ = 0.214) received weaker support, and the interaction model performed poorest (BF_10_ = 0.013). Inclusion Bayes Factors (all < 0.3) similarly provided little justification for adding these predictors. Overall, the Bayesian results are best characterized as inconclusive, indicating no reliable evidence for an effect of 5G exposure on SICI under the tested conditions (Figure 5C).

### 3.5. The Effects of 5G Mobile Phone EM Exposure on LICI

The two-way ANOVA showed no significant interaction between exposure condition (5 min, 20 min, sham) and time (Pre, Post) for LICI (*p* = 0.1887). Mean LICI values remained consistent across conditions, with no meaningful differences between exposures.

Bayesian analysis indicated that the null model had the highest posterior probability (*p* = 0.442), but the associated Bayes Factor (BF_10_ ≈ 1.00) was close to 1, reflecting inconclusive evidence. The time-only model showed a posterior probability of 0.282 (BF_10_ = 0.639), again indicating results close to the null and not strongly favoring any model. Models including exposure effects had substantially lower posterior probabilities (*p* < 0.20; BF_10_ = 0.152–0.289), and inclusion Bayes Factors (<0.3) suggested little justification for adding exposure or interaction terms.

Taken together, these findings provide inconclusive evidence and do not support a measurable effect of 5G exposure on LICI under the tested conditions (Figure 5D).

### 3.6. Summary of All TMS Measures

Statistical analysis confirms that 5G mobile phone EM exposure, between the three experimental conditions: 5 min exposure, 20 min exposure, and sham exposure, did not induce any statistically significant alterations in CSE (Figure 5A), ICF (Figure 5B), SICI (Figure 5C), and LICI (Figure 5D). This suggests that mobile phone EM exposure does not modulate CSE and intracortical excitability under the tested parameters.

## 4. Discussion

To our knowledge, this is the first in vivo study to use TMS to assess the impact of 5G mobile phone EM exposure on CSE, ICF, SICI, and LICI in healthy humans. Contrary to our initial hypothesis, short-term exposure (5 or 20 min) did not produce any significant changes in the assessed neurophysiological measures when compared with the sham condition. Although we expected that a more prolonged exposure might produce more pronounced effects, this duration-dependent modulation was not observed. This study addresses a gap in current research, which has mainly focused on lower-frequency EMR (such as 2G, 3G, 4G) and their effects on CSE. By examining excitatory and inhibitory mechanisms in the motor cortical, this work provides critical initial data for understanding the potential neurological effects of 5G technology, which is becoming more common worldwide. These findings support public health discussions by offering initial evidence that short-term exposure to 3.6 GHz EMR may not cause immediate changes in motor cortical excitability under the tested conditions. These findings contribute to ongoing public health and regulatory discussions, including those led by international agencies such as ICNIRP [47], IEEE [46], World Health Organization (WHO) [94], and ARPANSA [95], which continually review exposure limits, as well as broader community debates about the potential neurological and cognitive effects of 5G MP use. Bayes factors across all measures (CSE, ICF, SICI, LICI) were close to 1, indicating that the data were nearly equally consistent with both the null and alternative hypotheses. Accordingly, the lack of significant findings in the frequentist analysis should be interpreted with caution, as the Bayesian evidence is best described as inconclusive rather than confirmatory. In public health research, such inconclusive results emphasize the importance of replication and further testing, especially in longer or higher-intensity exposure scenarios.

### 4.1. Effect of 5G Mobile Phone EM Exposure on CSE

The primary aim of this study was to determine whether 5G EMR exposure at 3.6 GHz alters CSE, as assessed by the amplitude of MEPs induced by TMS. We observed no statistically significant differences between the active and sham conditions (*p* > 0.05), regardless of exposure duration. These findings align with previous studies that have reported no significant changes in MEP amplitude or intracortical inhibition after short-term RF-EMF exposure [48,49]. The Bayesian analyses provided additional perspective: while the null model had the highest posterior probability (*p* = 0.620; BF_10_ ≈ 1.00), Bayes factors close to 1 indicate that the data were nearly equally consistent with both the null and alternative hypotheses. Therefore, the evidence should be regarded as inconclusive rather than confirmatory. Inclusion Bayes factors were also low (BF_incl_ = 0.160–0.702), suggesting limited justification for adding exposure or time predictors. Together, these results indicate that under the tested conditions, short-term 5G EMR did not measurably alter corticospinal excitability, although subtle effects cannot be completely excluded.

A probable explanation is the inherent resilience of the cortex networks. The excitation–inhibition balance is precisely regulated through homeostatic plasticity and synaptic scaling, allowing GABAergic and glutamatergic systems to counteract minor external disturbances [96,97]. Furthermore, the exposure intensity in this study (power density = 0.0030 W/m^2^; E-field = 1.5 V/m) was well below international safety limits, likely insufficient to alter synaptic function. Exposure duration may also play a role: previous 3G/4G studies reporting changes in intracortical facilitation and inhibition used longer exposures (30–45 min), whereas our (5–20) minute exposures may have been too brief to elicit detectable neurophysiological effects. These conclusions are limited by the specific exposure durations of 5 and 20 min tested to mirror MP use. Longer or cumulative exposures might lead to different outcomes. Moreover, individual differences and mechanisms like homeostatic plasticity may have helped maintain baseline excitability [98]. Variations in study outcomes may stem from differences in exposure parameters, such as frequency, power, SAR, and duration. Demographic factors, such as age, sex, and cortical reactivity, also affect EMR susceptibility. For example, hormonal fluctuations [99,100,101] and age-related neuroplasticity [102,103,104] may modulate CSE and inhibitory control. Future research should stratify participants based on these variables to clarify sources of inter-study variability.

### 4.2. Effect of 5G Mobile Phone EM Exposure on ICF, SICI, LICI

To further investigate potential changes in CSE, we investigated the effects of 5G mobile phone EM exposure on ICF and inhibition (SICI, LICI), which are mediated by glutamatergic and GABAergic neurotransmission. No significant changes were observed in ICF following either exposure duration. This finding contrasts with that of [48], who reported increased ICF after 45 min of EMR exposure, potentially via modulation of NMDA receptors [105]. Conversely, our results are consistent with those of [49], which observed no ICF changes at lower frequencies (800 MHz), suggesting that frequency or intensity-specific mechanisms may underlie these differential effects.

Bayesian analysis showed that the time-only model (BF_10_ = 1.164) and the null model (BF_10_ ≈ 1.00) both yielded values close to 1, indicating that the data were nearly equally consistent with both the null model and the alternative hypotheses. This provides inconclusive rather than decisive evidence. Models involving exposure or its interaction with time had Bayes factors well below 1, indicating minimal explanatory value. Overall, these results suggest that under the present conditions, 5G exposure did not measurably affect NMDA receptor–mediated facilitation in the motor cortex.

Similarly, SICI, mediated by GABA-A receptor activity [71,106], was unaffected by 5G exposure. Previous studies have yielded mixed findings; [48] noted reductions in SICI, while others did not evaluate this outcome [49], leaving the evidence base inconclusive. The Bayesian analysis for SICI also produced Bayes factors near 1 (BF_10_ ≈ 1.00), indicating that the data did not provide meaningful evidence for either the null or alternative hypothesis. Inclusion Bayes factors were below 1 (BF = 0.543), showing little justification for adding exposure or time predictors. Taken together, the evidence remains inconclusive with respect to GABA-A–mediated inhibition under these conditions.

In the case of LICI, no significant changes were detected either. LICI is linked to GABA-B receptor function [36]. To date, this inhibitory mechanism has not been extensively examined in the context of EMR exposure, thereby positioning our study as one of the few investigations of it in this setting. Bayesian results for LICI again showed Bayes factors close to 1. The time-only model had a Bayes Factor of 0.639, which does not provide meaningful evidence for a time effect. Overall, the Bayesian results remain inconclusive, indicating no measurable influence of 5G exposure on GABA-B–mediated inhibition under the tested conditions.

From a neuroscience perspective, one explanation for the observed null results across CSE, ICF, SICI, and LICI may lie in the neurobiological resilience of cortical circuits. Acute exposure to low-level RF-EMF might not reach the threshold required to alter synaptic efficacy within motor cortical networks, especially considering the known homeostatic mechanisms that tightly regulate excitation–inhibition balance. Cortical GABAergic and glutamatergic systems are capable of adapting to transient perturbations through plasticity or feedback inhibition, thereby maintaining baseline excitability. Our findings are consistent with recent EEG studies that observed minimal or inconsistent effects of 3G/4G/5G exposure on neural oscillations, particularly in the alpha and beta bands. These studies suggest that although EM exposure may subtly affect network-level synchronization, it may not translate into measurable changes in CSE or intracortical excitability using TMS. Furthermore, discrepancies between our results and prior TMS-based studies may reflect differences in RF frequency, SAR levels, stimulation site, and even methodological rigor, such as randomization or considering different exposure distances. These results suggest that, at the tested frequency and duration, short-term 5G EMR does not alter excitatory or inhibitory intracortical circuits. However, future work should explore these outcomes under varied exposure intensities and durations.

### 4.3. Limitations and Suggestions for Future Research

This research provided valuable insights into the effects of 5G mobile phone EM exposure at a frequency of 3.6 GHz on CSE and related mechanisms such as ICF, SICI, and LICI. However, several limitations deserve attention. This study did not account for differences between mobile phone models or operating systems (e.g., Android vs. Apple), which can affect radiation levels through variations in hardware, antenna design, and power output. A key limitation is the reliance on a single 5G phone model, specifically the Galaxy A13 (SM-A136B). Although this device is widely used and provides a practical basis for controlled testing, differences in antenna configuration, SAR values, and network settings across devices may alter output power and exposure characteristics. These differences could influence the consistency and applicability of the results, as other devices may create unique exposure conditions [107]. The relatively small sample size may limit the study’s statistical power. Therefore, the findings should be considered exploratory rather than confirmatory [60,61]. The discrepancy between the a priori power calculation and the actual number of recruited participants underscores the pilot nature of the work. This research was not designed to achieve the statistical power required for hypothesis testing; instead, it aimed to refine methodological procedures and generate preliminary effect size estimates to guide the design of future adequately powered trials [61,63]. Additionally, the study did not examine the impact of gender factors, including the effects of gender and age. Evidence suggests that sex hormones such as Estrogen and Androgen can influence cortical excitability and neuroplasticity, potentially leading to different responses in men and women [99,100,102]. The current research hypothesizes that women, particularly during periods of high Estrogen levels, may show different neuroplasticity and altered responses compared to men [101,108].

The experimental outcomes may vary in older adults, whose cortical excitability and adaptability undergo age-related modulation [104]. The timing of assessments is a limitation. We measured CSE and intracortical excitability after exposure to avoid EMR interference between TMS systems and the active 5G handset, while prioritizing participant safety and comfort [68,69,70]. This study focused solely on short-term exposure. Long-term follow-up assessments involving different SAR and PD levels are necessary to evaluate potential cumulative or delayed effects. Lastly, the scope of the study was limited to motor cortical measures. Possible impact on cognitive functions, such as attention, working memory, and executive control, as well as on sensory processing (e.g., visual and auditory perception), remains unexplored.

Another limitation of this study is the short exposure durations (5 and 20 min), which were selected to reflect typical real-life mobile phone use but may not have been sufficient to induce measurable changes in CSE. Our hypothesis was exploratory, anticipating that even brief exposure to 5G MP might modulate cortical excitability. However, both frequentist and Bayesian analyses yielded inconclusive evidence, with Bayes factors close to 1 indicating that the data were equally consistent with the null and alternative models. Thus, our findings should be interpreted as showing no detectable change under the tested conditions, rather than definitive evidence of no effect.

In conclusion, future studies are necessary to establish standardized comparisons across various MP models and operating systems, thereby reducing device-related variability. Future studies should recruit larger, stratified samples that encompass both sexes and a wider age range to capture gender and age-dependent effects, not only in healthy participants but also in vulnerable populations. In such groups (e.g., children, older adults, or individuals with neurological or psychological conditions), systematic safety verification, using personal dosimetry and basic clinical assessments, will be essential.

Additionally, future investigations could incorporate protocols that examine longer exposures and include real-time monitoring during active exposure to better assess potential delayed or chronic outcomes. Since all assessments were conducted only after exposure to avoid interference and ensure safety, the study was unable to capture real-time effects. Future work should develop safe methods for measuring neurophysiological responses during active 5G exposure. Lastly, further research should expand beyond motor cortex measures to include cognitive and sensory domains, allowing for a more comprehensive understanding of the effects of 5G EMR on CSE and intracortical excitability.

Finally, it is important to consider the broader public health context of these findings. The exposure levels recorded in this study (0.0030 W/m^2^; 1.5 V/m) were significantly below international safety limits (ICNIRP/IEEE: 40 W/m^2^). While this suggests that our null results are reassuring for everyday short-term 5G exposure scenarios, it also emphasizes the need for further research under longer-term, higher-intensity, or cumulative exposure conditions to fully understand potential public health implications.

## 5. Conclusions

This pilot study contributes to our understanding of 5G mobile phone EM exposure (3.6 GHz) on cortical excitability. Short-term exposure (5 or 20 min) did not induce detectable changes in CSE, ICF, SICI, or LICI compared with sham conditions, as assessed by TMS. Both frequentist and Bayesian analyses supported the absence of measurable effects under these experimental conditions. While these findings suggest that short-term 5G exposure is unlikely to induce robust neurophysiological alterations, they must be interpreted within the limitations of this pilot design. Future studies should investigate longer exposure durations, higher power densities, and alternative frequencies to determine whether more subtle or cumulative effects may emerge.

## Figures and Tables

**Table 1:** A summary of TMS studies investigating the effects of mobile phone EM exposure on Corticospinal Excitability (CSE), Intracortical Facilitation (ICF), Short-interval Intracortical Inhibition (SICI), and Long-Interval Inhibition (LICI).

(Study)	Participants’ Gender and Age Range (Mean)	Mobile Phone Specifications (Frequency, Power, SAR)	Exposure Time	Study Design (Crossover/Blinding)		TMS Device Specifications	Observed Effects			
				Single Blind	Double Blind		CSE	ICF	SICI	LICI
Inomata-Teradal., 2007 [49]	10 (5 M, 5 F) 22–51 Y	800 MHz/ 270 mW	30 min	_	√	200 (The Magstim Co., Ltd., Whitland, UK)	_	↑	N/S	N/S
Ferreri, F., et al., 2006 [48]	15 F 20–36 Y	902.40 MHz/ 2 W	45 min	_	√	200 (The Magstim Co., Dyfed, UK)	_	↑	↓	N/S

Note: M: Male; F: Female; Y: Years old; F: Frequency; SAR: Specific Absorption Rate; MHz: Megahertz; √: Yes: W: Watt; ↑ = Increased, ↓ = Decreased, _ = No significant changes; N/S = Not Studied.

## Data Availability

The data presented in this study are available on request from the corresponding author due to ethical restrictions and participant confidentiality.
